# Molecular detection of *Mycobacterium tuberculosis* sensitivity to rifampicin and isoniazid in South Gondar Zone, northwest Ethiopia

**DOI:** 10.1186/s12879-019-3978-3

**Published:** 2019-04-25

**Authors:** Amir Alelign, Aboma Zewude, Temesgen Mohammed, Samuel Tolosa, Gobena Ameni, Beyene Petros

**Affiliations:** 1 Aklilu Lemma Institute of Pathobiology, Addis Ababa University, P.O. Box 1176, Addis Ababa, Ethiopia; 2 College of Natural Sciences, Department of Microbial, Cellular and Molecular Biology, Addis Ababa University, P.O. Box 1176, Addis Ababa, Ethiopia; 3 College of Natural and Computational Sciences, Department of Biology, Debrebrhan University, P.O. Box 445, Debrebrhan, Ethiopia

**Keywords:** *M. tuberculosis*, Drug sensitivity, South Gondar, Northwest Ethiopia

## Abstract

**Background:**

Drug resistant tuberculosis (TB) has become a persistent health threat in Ethiopia. In this respect, baseline data are scarce in many parts of high TB burden regions including the different zones of Ethiopia.

**Methods:**

A total of 111 culture positive *M. tuberculosis* isolates were recovered from TB patients and identified using region of difference (RD) 9 based polymerase chain reaction (PCR) and spoligotyping. Thereafter, their drug sensitivities to Rifampicin (RIF) and Isoniazid (INH) were evaluated using GenoType MTBDR*plus* assay**.**

**Results:**

The result showed that 18.0% (20/111) of the isolates were resistant to either RIF or INH. Furthermore, 16.7 and 23.8% of the isolates from new and retreatment cases were resistant to any of the two anti-TB drugs, respectively. Multi-drug resistant (MDR) TB was detected on 1.8% (2/111) of all cases. Significantly higher frequencies of any drug resistance were observed among Euro-American (EA) major lineage (χ^2^: 9.67; *p* = 0.046).

**Conclusion:**

Considerably high proportion of drug resistant *M. tuberculosis* strains was detected which could suggest a need for an increased effort to strengthen TB control program in the study area.

## Background

Tuberculosis (TB) is an infectious disease caused by *M. tuberculosis* complex and persists to be a public health threat globally. In 2016, there were an estimated 10.4 million incident cases and about 25% of which were inhabitants of Africa [1]. In Ethiopia, the estimated total TB incidence in 2016 was 177 per 100,000 population. This was greater than the global average of 140 /100,000 [1].

Drug resistant TB remains to be a public health threat in many parts of the world. About 490,000 cases of multidrug resistant-TB (MDR-TB) and an additonal 110, 000 cases that were susceptible to INH but resistant to RIF, RR-TB; the most effective first-line anti-TB drug were reported at the end of 2016 [1]. According to the same report, an estimated 4.1% of new cases and 19% of previously treated cases had MDR/RR-TB. In this respect, Ethiopia has reported a relatively lower level of drug resistant-TB (DR-TB) cases than the global average, 2.7% resistance in newly diagnosed and 14% of previously treated cases, respectvely [1].

Many separate studies in Africa suggested the public health risk of drug resistant-TB. A study conducted in Addis Ababa, Ethiopia, reported 72.9% of the *M. tuberculosis* isolates were resistant to at least one of the first-line anti-TB drugs [2]. A similar study in Oromia region of the country, reported 33% of the cases were MDR-TB [3]. Studies from other parts of Africa such as South Africa, Zambia, Zimbabwe, Uganda, Tanzania and Kenya reported significant proportions of *M. tuberculosis* isolates were resistant to at least one of the first-line anti-TB drugs [4–9].

In the Amhara region of northwest Ethiopia, drug resistance remains to be one of the major obstacles to the effort towards effective TB control. Previous studies in the region suggested a high (ranging from 15.8 to 33.5%) resistance level of *M. tuberculosis* for at least one of the currently available first-line anti-TB drugs which was affected by different patient and microbial characteristics [10–12]. However, no enough data is available in this respect in different zones of the region. Therefore, the present study was conducted to determine anti-TB drug resistance and its associated risk factors in South Gondar Zone of the Amhara Region, northwest Ethiopia.

## Methods

### The study area

The study was conducted in South Gondar Zone of the Amhara Region, northwest Ethiopia. Debre Tabor is the seat of the administration of the Zone and is located at 666 km from Addis Ababa in the northwest direction and at 99 km from Bahir Dar City in the same direction. The Zone consisted of 10 districts and inhibited by 2,051,738 people. The population density of the Zone was 145.56 per square km; 90.47% of its population was living in the rural area. The total number of households in the Zone were 468,238 and average number of persons per a household was 4.38 [13]. The livelihood of the community largely depended on subsistence agriculture [14].

### Study design and participants

A cross-sectional study was conducted between March 2015 and April 2017 to determine anti-TB drug sensitivity of *M. tuberculosis* isolated from TB cases in the South Gondar Zone. All forms (pulmonary and extra pulmonary) TB cases who visited the health centers, zonal and regional referal hospitals were included in this study. All extra pulmonary cases were TB lymphadenitis. TB patients who were under treatment prior to the start of the study and those under 5 years of age were excluded from the study.

### Data collection and processing

After informed consent was obtained from participants, sputum samples and fine needle aspirate (FNA) samples were collected by trained laboratory technicians and pathologists.

The samples were first digested and concentrated/decontaminated by the N-acetyl-L-cysteine-Sodium hydroxide (NALC-NaOH) method. Smears of the final deposits from the various specimens were stained by the Ziehl-Neelsen (ZN) method and examined under oil immersion using a binocular light microscope. All smear positive TB samples were stored at + 4 °C at the study site and then transported to Regional Health Research Llaboratory Center (Bahirdar) and kept at + 4 °C until bacteriological culture was performed.

FNA samples were collected by pathologsts using standard needle and syringes and stored in cryo-tubes with phosphate buffer saline (PBS) pH 7.2. ZN staining was used to detect acid-fast/ tubercule bacilli (AFB). The AFB-positive samples were stored in a similar way to that of sputum samples for further investigations.

### Mycobacterium culture

The samples were processed for culturing according to the standard methods described earlier [15, 16]. Both sputum and FNA samples were cultured at the Bahir Dar Regional Health Research Laboratory Centre. Briefly, about 100 μl of the sample suspension was inoculated on four sterile Lowenstein Jensen (LJ) medium slopes (two were supplemented with pyruvate and the other two with glycerol) and then incubated at 37 °C with weekly examination for growth. Cultures were considered to be negative when no visible growth was detected after eight weeks of incubation. The presence of AFB in positive cultures was detected by smear microscopic exa-mination using the ZN staining method. AFB positive cultures were prepared as 20% glycerol stocks and stored at − 80 °C as reference. Heat-killed cells of each AFB isolate were prepared by mixing ~ 2 loopfuls of cells (≥ 20 μl cell pellet) in 200 μl distilled H_2_O followed by incubation at 80 °C for 45 min for the release of DNA after the breaking the cell wall. The heat killed cells were transported to the laboratory at Aklilu Lemma Institute of Pathobiology, Addis Ababa University, for molecular typing.

### Region of difference (RD) 9-based polymerase chain reaction

To differentiate *M. tuberculosis* from other members of the *M. tuberculosis* complex (MTBC) species, RD9 based PCR was performed according to protocols previously described [17].

*M. tuberculosis* H37Rv, *M. bovis* bacille Calmette-Guérin (BCG) were included as positive controls and water was used as a negative control. Interpretation of the result was based on bands of different sizes (396 base pairs (bp) for *M. tuberculosis* and 375 bp for *M. bovis*) as previously described [18].

### Spoligotyping

Isolates genetically identified as *M. tuberculosis* were spoligotyped for further strain identification. Spoligotyping makes use of the variability of the MTBC chromosomal direct repeat (DR) locus for strain differentiation as previously described [19]. A PCR-based amplification of the DR region of the isolate was performed using oligonucleotide primers derived from the DR sequence. The amplified product was hybridized followed by subsequent membrane washing processes. Known strains of *M. bovis* and *M. tuberculosis* H37Rv were used as positive controls, whereas Qiagen water (Qiagen Company, Germany) was used as a negative control. Hybridized DNA was detected by the enhanced chemiluminescence method. The presence or absence of spacer was used as key for the interpretation the result.

### Identification of *M. tuberculosis* strains and lineages

A web-based spoligotype database was utilized to assign shared international types (SITs) for the isolates [20]. Strains matching a preexisting pattern in the database were identified with SIT number otherwise considered as orphans. An online TB lineage tool was used to predict the major lineages using a conformal Bayesian network (CBN) analysis and sub lineage/family using knowledge based Bayesian network (KBBN) [21].

### Drug sensitivity test (DST) by molecular method

The GenoType MTBDR*plus* assay which is a molecular genetic assay was used to detect resistant *M. tuberculosis* isolates to RIF and INH. The assay is based on the DNA-STRIP technology. The whole procedure involves a multiplex PCR amplificaton with biotinylated primers, and a reverse hybridization. This assay detects for the abscence and/or presence of wild type (WT) and/or mutant (MUT) DNA sequences with in specific region of three genes: the rpoB gene (coding for the β-subunit of the RNA polymeraze), for the identification of RMP resistance; the katG gene (coding for the catalase peroxidase), for high level INH resistance; and the promoter region of the inhA gene (coding for the NADH enoylACP reductase), for low-level INH resistance. The procedure of the test was performed based on the manufacturer’s instructions (Hain Life sciences, Nehren, Germany).

### Data analysis

Data were double entered to Excile file format and statistical analysis was performed using SPSS software version 25. Descriptive statistics were used to depict the demographic variables. Bivariate logistic regression analysis was performed to obtain crude odds ratio (COR) and the corresponding 95% confidence interval (CI) to assess the association of risk factors and the odds of drug sensitivity. The Pearson Chi-square (χ^2^) was calculated to test the association of drug resistance with genotype of *M. tuberculosis* isolates from TB patients. Results were considered statistically significant whenever *p*-value was less than 5%.

## Results

### Drug resistance patterns among the different patient characteristics

The drug sensitivity test was performed for 111 culture positive *M. tuberculosis* isolates obtained from both pulmonary and extra pulmonary TB patients. Of which, 91 (82.0%) were susceptible to both RIF and INH and 20 (18.0%) were resistant to atleast one of the two drugs. Of the total 20 drug resistant isolates, the male to female ratio was 1:1. Younger adults aged between 18 and 30 years constituted about 45% (9/20) of the total drug resistant cases (see Table 1).

**Table 1:** Socio-demographic characteristics of study participants and their association with drug sensitivity patterns of *M. tuberculosis* isolates, South Gondar Zone, Amhara region, northwest Ethiopia (2015–2017)

Characteristics	Any drug resistance			COR (95% CI)	*p*-value
	Yes, n (%)	No, n (%)	Total, N (%)		
Sex					
Male	10 (17.5)	47 (82.5)	57 (100)	1.00	
Female	10 (18.5)	44 (81.5)	54 (100)	1.06 (0.40–2.81)	1.00
Age (years)					
5–17	2 (18.2)	9 (81.8)	11 (100)	1.00	
18–30	9 (21.4)	33 (78.6)	42 (100)	1.22 (0.22–6.72)	0.813
31–43	3 (8.8)	31 (91.2)	34 (100)	0.43 (0.06–3.02)	0.390
44–56	4 (22.2)	14 (77.8)	18 (100)	1.28 (0.19–8.53)	0.794
> 56	2 (33.3)	4 (66.7)	6 (100)	2.25 (0.22–22.14)	0.481
Patient category					
New cases	15 (16.7)	75 (83.3)	90 (100)	1.00	
Retreatment	5 (23.8)	16 (76.2)	21 (100)	1.56 (0.49–4.92)	0.443
Family TB history					
Yes	6 (16.7)	30 (83.3)	36 (100)	1.00	
No	14 (18.7)	61 (81.3)	75 (100)	1.14 (0.40–3.28)	0.797
Clinical presentation					
PTB	11 (25.0)	33 (75.0)	44 (100)	1.00	
EPTB	9 (13.4)	58 (86.6)	67 (100)	0.46 (0.17–1.23)	0.120

*COR* crude odds ratio, *CI* confidence interval

Of 90 newly diagnosed TB cases, 75 (83.3%) were susceptiable to both RIF and INH (RIFs, INHs) but the remaining 15 (16.7%) were INH mono-resistant (RIFs, INHr). Similarly, 23.8% (5/21) of the retreatment cases were resistance to either of RIF and INH (see Table 1).

Despite the observed differences in the proportions of drug resistant cases, no statistically significant association was observed between demographic and clinical characteristics of patients with drug resistance. From a total of 21 retreatment TB cases, 76.2% (16/21) were suscebtible to both drugs and the remaining 23.8% (5/21) were resistant to atleast one of the two anti-TB drugs (see Table 1).

MDR-TB cases (resistance to both RIF and INH) were observed in 1.8% (2/111) of the cases. Both cases were detected from retreatment TB patients. No RIF mono-resistant (RIFr, INHs) case was detected in any of disease or patient categories (see Table 2).

**Table 2:** Resistance pattern of *M. tuberculosis* isolates to INH and RIF with TB patient category, South Gondar Zone, Amhara region, northwest Ethiopia (2015–2017)

Drug resstance pattern	New cases (%) (*n* = 90)	Retreatment (%) cases (*n* = 21)	Total (%) (*N* = 111)
Any Susceptible	75 (83.3)	16 (76.2)	91 (82.0))
Any resistance	15 (16.7)	5 (23.8)	20 (18.0)
INHmono- resistance	15 (16.7)	3 (14.3)	18 (16.2)
INH Susceptible	75 (83.3)	16 (76.2)	91 (82.0)
RIF mono-resistance	0 (0.0)	0 (0.0)	0 (0.0)
RIF Susceptible	90 (100)	19 (90.5)	109 (98.2)
MDR (INH + RIF resistance)	0 (0.0)	2 (9.5)	2 (1.8)
Hetroresistance	6 (6.7)	0 (0.0)	9 (8.1)

*INH* Isoniazid, *RIF* Rifampicin, *MDR* Multidrug resistance

### Drug resistance confering gene mutations

From the total samples tested for drug sensitivity, 18.0% (20/111) of them showed atleast one gene mutation. Among the three targeted genes (*katG*, *rpoB* and *inhA*), mutations were observed only on the *katG* and *rpoB* genes, confering a high level of INH resistance and RIF resistance, respectively. All INH-resstance confering mutations occured only at *katG* gene (*katG* MUT1). In the present study, no *inhA* gene mutaion was observed. About 1.8% (2/111) RIF-resistance confering gene mutations occured and resulted from the absence of *rpoB* WT7, without the presence of MUT3 in both specimens. Both the RIF-resistance confering gene mutations at *probB* occured together with INH-resistance confering gene mutations at *katG* genes, signaling MDR-TB pattern. Among the 20 INH-resistance confering mutations, specific mutations (the WT probe was missed with the presence of the corresponding MUT1 probe) of the *katG* gene were observed in 12 isolates. In 6 INH-resistant isolates, unexpected mutation patterns were observed, both the WT and the corresponding MUT1 probes of the *katG* gene were positive signaling a hetroresistance pattern and considered as ‘rare’ mutations (see Table 3). WT7 probes of the *proB* gene were missed without the presence of the corresponding MUT probes and considered as ‘unknown’ mutations (see Table 3).

**Table 3:** Distribution of target gene mutations and resistance patterns in anti-TB drug resistant *M. tuberculosis* isolates, South Gondar Zone, Amhara region, northwest Ethiopia (2015–2017)

		Target genes			
Isolates	*rpoB*	*rpoB katG*	*lnhA*	Type of mutation	Drug resistance pattern
FE31	WT1–8	MUT 1	WT1,2	specific	RIFs INHr
FE34	WT1–8	MUT1	WT1,2	specific	RIFs INHr
FE50	WT1–8	WT, MUT 1	WT1,2	rare	RIFs INHr^a^
FE51	WT1–8	MUT1	WT1,2	specific	RIFs INHr
FE54	WT1–8	MUT1	WT1,2	specific	RIFs INHr
FE55	WT1–8	MUT1	WT1,2	specific	RIFs INHr
FE56	WT1–8	WT, MUT1	WT1,2	rare	RIFs INHr^a^
FE72	WT1–8	WT, MUT1	WT1,2	rare	RIFs INHr^a^
FE74	WT1–8	MUT1	WT1,2	specific	RIFs INHr
FP12	WT1–8	WT, MUT1	WT1,2	rare	RIFs INHr^a^
WHC02	WT1–8	MUT1	WT1,2	specific	RIFs INHr
WHC13	WT1–8	MUT1	WT1,2	specific	RIFs INHr
WHC14	WT1–8	MUT1	WT1,2	specific	RIFs INHr
WHC405	WT1–8	WT, MUT1	WT1,2	rare	RIFs INHr^a^
WHC322	WT1–6, 8,	MUT1	WT1,2	unknown	RIFr INHr^b^
WHC710	WT1–6, 8,	MUT1	WT1,2	unknown	RIFr INHr^b^
AZ05	WT1–8	WT, MUT1	WT1,2	rare	RIFs INHr^a^
AZ06	WT1–8	MUT1	WT1,2	specific	RIFs INHr
WHN02	WT1–8	MUT1	WT1,2	specific	RIFs INHr
255A	WT1–8	MUT1	WT1,2	specific	RIFs INHr

^*a*^*Hetrores*istance, ^b^Multidrug resistance

*WT* Wild type, *MUT* Mutant type; the subscripts ‘s’ and ‘r’ refer to: sensitive and resistance, respectively

### RD9 deletion typing patterns of *M. tuberculosis* strains

A total of 106 culture-positive mycobacterial isolates were tested by RD9-based PCR. The test revealed that all isolates had intact RD9 locus and were subsequently classified as *M. tuberculosis.* No other species of MTBC was detected.

### Spoligotype patterns of *M. tuberculosis* strains

The patterns of 96 isolates were interpretable and grouped into 38 different spoligotype patterns. From the 38 patterns, 22 were shared types and consisted of 76 isolates. The remaining 16 were not registered in the database and were ‘orphans’ strains. The dominantly identified SITs were SIT53, SIT149, and SIT428, each consisting of 18, 12 and 12 isolates, respectively. These three SITs consisted of 43.8% of the total isolates.

Five major lineages were identified including Euro-American (EA), East-African-Indian (EAI), *M. africanum*, Indo-Oceanic (IO), and East-African-Asian (EAS), each consisting of 65.5, 26, 8.3, 2.1 and 1% of the isolates, respectively. The three dominantly identified families were T, CAS and Manu, each consisting of 46.9, 24.0 and 10.4% of the isolates, respectively.

### Association of drug resistance pattern and *M. tuberculosis* lineages

The majority of resistant strains for any of anti-TB drugs (RIF, INH, RIF and INH) were belong to the EA major lineage, 16.7% (16/96), sub-lineage T,10.4% (10/96), orphan dominant strains, 6.3% (6/96) and clustered strains, 13.5% (13/96). The two MDR cases belonged to the EA major lineage and T3-ETH sub-lineage.

A statistically significant association (χ^2^: 9.67; *p* = 0.046) was observed between any anti-TB drug resistance and major lineages of *M. tuberculosis* strains. Although the statistical association between the categories of sub-lineages, dominant strains and clustering level with any drug resistance of the isolates was not significant, differences in proportion were observed (see Table 4).

**Table 4:** Association of drug resistance with genotype of *M. tuberculosis* isolates from TB patients, South Gondar Zone, Amhara region, northwest Ethiopia (2015–2017)

Characteristics	Variables	Any drug resistance		Total	χ^2^(df)	*p*- value
		Yes	No			
Major lineage	EA	16	43	59	9.67 (4)	0.046
	EAI	0	25	25		
	EAS	0	1	1		
	IO	0	2	2		
	*M. africanum*	1	7	8		
Sublineages/clades	AFRI	0	7	7	14.1 (8)	0.080
	Beijing	0	1	1		
	CAS	0	23	23		
	H	1	6	7		
	Manu	3	7	10		
	T	10	20	30		
	T3-ETH	4	10	14		
	Turkey	0	3	3		
	X	0	1	1		
Dominant strains	Orphan	6	14	20	4.30 (3)	0.23`
	SIT53	4	14	18		
	SIT149	3	9	12		
	SIT428	0	12	12		
Clustering	No	5	20	25	0.34 (1)	0.852
	Yes	13	58	71		

*EA* Euro-American, *EAI* East-African-Indian, *IO* Indo-Oceanic, *EAS* East-African-Asian, *CAS* Central Asian Strain, *H* Harlem, *AFRI* African, *SIT* shared international types. χ^2^ Chi-square, *df* degree of freedom

## Discussion

In the present study, an over all resistance of 18.0% (20/111) was detected for either of RIF and INH. This was comparable with a previous study conducted in Southwest Ethiopia [22]. Similarly, an 18.4% [23] and 17.8% [12] resistance levels were reported in Eastern and northeastern Ethiopia, respectively. However, lower resistance levels to one of the first line anti-TB drugs were reported in the northwest (15.3%) [10] and northern (6.7%) [24] parts of the country, respectively. In contrast, higher resistance levels (ranged from 22 to 35.5%) than the present study were reported in different parts of the country [2, 11, 25–27]. Higher anti-TB resistance was also reported in some other East African countries such as Kenya (30%) and Rwanda (43%) [28, 29]. The observed differences in the level of anti-TB drug resistance between this study and previous studies might be due to dfferences in study settings, study design and sample size. Most of our study particpants were all forms of TB patients from rural areas and the majority (81.1%) of the study subjects were newly diagnosed, which might lower the intensity and transmission of drug resistant TB as compared to the urban and prison settings in the previous studies where higher chance of TB circulation and lower anti-TB drugs adherence could be noticed.

The 16.2% (18/111) INH monoresistance recorded in the present study was higher than those reported in other parts of Ethiopia [30, 31]. Lower prevalence of INH monoresistance was also reported in other East African countries such as Kenya (3.1%) and Tanzania (6.3%) [9, 32]. Similarly, lower levels of INH monoresistance cases were reported in Pakistan (6.3%) and south India (6.9%) [33, 34]. In contrast, higher levels of INH monoresistance were reported by other studies in Kenya (30.2%), Ethiopia (27.7%) and Guinea (18%) [28, 35, 36]. Repeated use of the drug and patients lower adherance to the antibiotc might be responsible for the overall higher INH monoresistance level in the present study.

In this study, the low prevalences (1.8%) of MDR-TB was comparable with previous studies conducted in Ethiopia [26, 27, 37]. However, higher levels of MDR-TB cases were revealed in Oromia region (33%) and southwest Ethiopia (27.7%) [3, 38]. Studies conducted in Uganda (14%) and Tanzania (6.3%) reported higher frequencies of MDR-TB cases among new TB cases [5, 7]. All the above investigations were cross-sectional studies but the dfferences in overall prevalence of MDR-TB might be due to variation in the number of samples and selection of the study subjects. Samples from TB-specialized hospitals and the majority of retreatment cases could increase the number of MDR-TB cases unlike the present study where the majority samples were from rural dwellers in government health centers and peripheral health posts and about 81% of the study subjects were newly diagnosed for TB.

In the present study, we observed a complete preference of *katG* mutations without concurrent *inhA* promoter mutation. All (20/111) the anti-TB drug resistant strains were resistant to INH, and all the INH-resistance had resulted because of mutations at *katG* gene. In agreement to our findng, a study conducted in another part of the Amhara region reported that all the INH-resistant isolates were due to mutations at *katG* gene [24]. Higher frequencies of *katG* mutations were also reported in other parts of Ethiopia [23, 38, 39]. All the above facts suggested the emergence of high level INH-resistnce among *M. tuberculosis* strains circulating in the study area which potentially could compromise TB treatment and control.

In our study, exceptions to specifc mutation patterns were observed. In six INH-resistant *M. tuberculosis* isolates, unusual or rare mutation pattern was noticed at *katG* gene in which both the *katG*WT and *katG*MUT types were present simultaneously, signaling hetroresistance condition. Moreover, in the two MDR-TB cases, the WT7 probes of the *proB* gene were missed without the presence of the corresponding MUT probes and considered as ‘unknown’ mutations. Both these unexpected mutation patterns were also reported in several studies in Ethiopia [38, 39]. The hetroresistant cases might be due to selection of cells with random mutatons during inadequate treatment or by mixed infections with sensitive and resistant cells. This hetroresistance phenomenon will jeoparadize the effective treatment of patients with INH there by leading to the development of anti-TB drug resistance in the study area.

In this study, any drug resistance to RIF and INH was not found to be proportionally distributed among *M. tuberculosis* genotypes. However, a statistically significant association was observed between any types of anti-TB drug resistance and EA lineage. Similarly, a higher frequency of any drug resistance to first line anti-TB drugs among EA lineage of *M. tuberculosis* was reported in central Ethiopia [27]. Although the association was not statistically significant, the observed highest proportion of drug resistant cases among the T-sublineage investgated in this study was similar to studies from other African countries, Tanzania [32] and Uganda [40]. In this study, clustered strains showed higher frequency of any anti-TB drug resistance, suggesting higher risk of drug resistance in recent TB transmission in the study area.

## Conclusions

Considerable proportion of *M. tuberculosis* strains was found to be resistant to at least one of RIF and INH. Significant variatons were observed in drug resistance patterns among *M. tuberculosis* lineages. Therefore, increased efforts in timely evaluatlon of drug resistance and application of more roboust diagnostic tools may help in the implementation of effective TB control program in the study area.

### Limitations

Although our study wass the first report concerning the gentetic basis of drug resistance and its association of genotypes among *M. tuberculosis* isolates circulatng in south Gondar zone, northwest Ethiopia, it had some limitations. The molecular detection of drug resistance among *M. tuberculosis* isolates was not compared with the routinely used conventional drug sensitivity method. In addition, molecular characterization of mycobacterial strains was not conducted using better molecular diagnostic tools than spoligotypng.

## Abbreviations

AFB: Acid-fast bacilli
CBN: Conformal Bayesian network
EA: Euro-American
EAI: East-African-Indian
EAS: East-African-Asian
FNA: Fine needle aspirate
INH: Isoniazid
IO: Indo-Oceanic
KBBN: Knowledge based Bayesian network.
LJ: Lowenstein- Jensen
MDR: Multi-drug resistance
MIRU: Mycobacterial interspersed repetitive units
MTBC: *Mycobacterium tuberculosis* complex
MUT: Mutant
PBS: Phosphate buffer saline
PCR: Polymeraze chain reaction
RD: Region of difference
RIF: Rifampisin
RR: Rifampisin resistance
SIT: Spoligotype International Type
TB: Tuberculosis
WT: Wild type
XDR: Extensively drug-resistant
